# Surgical Risk Following Carpal Tunnel Release in Non-cirrhotic Non-alcoholic Fatty Liver Disease Versus Non-alcoholic Cirrhosis: A Propensity-Matched Analysis

**DOI:** 10.7759/cureus.103871

**Published:** 2026-02-18

**Authors:** Catherine Hand, Jared Sasaki, Francis Hand, Daniel Shinn, Nicholas Lemme, Brian Forsythe

**Affiliations:** 1 School of Medicine, University of Texas Health Science Center at San Antonio, San Antonio, USA; 2 School of Medicine, New York Medical College, Valhalla, USA; 3 Department of Orthopedic Surgery, Rush University Medical Center, Chicago, USA; 4 Orthopaedic Surgery, Midwest Orthopaedics at Rush, Chicago, USA

**Keywords:** carpal tunnel release, hand surgery, non-alcoholic cirrhosis, non-alcoholic fatty liver disease, postoperative complications

## Abstract

Background

Non-alcoholic fatty liver disease (NAFLD) and its progression to non-alcoholic cirrhosis (NAC) frequently coexist with metabolic and musculoskeletal conditions, including carpal tunnel syndrome. While cirrhosis is a known risk factor for adverse surgical outcomes, its effect on low-acuity orthopedic procedures such as carpal tunnel release (CTR) remains unclear. This study compares perioperative outcomes following CTR in patients with NAFLD versus NAC.

Methodology

This retrospective cohort analysis screened 334,595 CTR patients, from which 1,516 with NAC and 6,427 with NAFLD were identified. After one-to-one matching based on age, gender, Charlson Comorbidity Index (CCI), and key comorbidities, 642 patients per group were analyzed. In this study, 90-day and one-year postoperative complications were identified using the International Classification of Diseases and Current Procedural Terminology codes. Multivariable logistic regression models adjusted for age, sex, and CCI. Cost outcomes were evaluated using both the Wilcoxon rank-sum and t-tests comparing 90-day reimbursement data between matched cohorts.

Results

Patients with NAC had significantly higher rates of deficiency anemia and lower rates of obesity compared to those with NAFLD. No statistically significant differences were observed in 90-day complications, but at one year, cirrhotic patients were more likely to develop acute kidney injury (AKI). Cost analysis showed that patients with NAC had higher mean 90-day reimbursements than those with NAFLD, though the difference was not statistically significant.

Conclusions

Patients with NAC experienced a higher risk of AKI at one year compared to those with NAFLD, but this may be attributed to hepatorenal syndrome that cirrhotic patients experience. These findings suggest that hepatic disease severity may not significantly influence postoperative outcomes, and patients with more advanced liver pathology may not require increased perioperative surveillance compared with those with NAFLD following CTR.

## Introduction

Carpal tunnel release (CTR) is one of the most frequently performed hand procedures in the United States, with lifetime incidence rates estimated at 1.9% for men and 4.1% for women [1]. Although CTR is generally considered a low-risk procedure, the influence of systemic conditions such as hepatic disease on postoperative outcomes remains underexplored in the current literature [2].

Non-alcoholic fatty liver disease (NAFLD) or metabolic dysfunction-associated steatotic liver disease, affecting approximately 30% of adults worldwide, represents a spectrum of hepatic pathology that ranges from simple steatosis to non-alcoholic steatohepatitis (NASH) and eventually cirrhosis [3]. With the rising prevalence of obesity and metabolic syndrome, NAFLD is now the leading cause of chronic liver disease in Western countries [3,4]. In contrast, non-alcoholic cirrhosis (NAC) represents an advanced stage with fibrotic remodeling and portal hypertension, conferring increased systemic and surgical risk [5].

Patients with cirrhosis have demonstrated increased morbidity and mortality following abdominal, orthopedic, and cardiovascular procedures [6,7]. However, the impact of liver disease severity on ambulatory procedures such as CTR remains underexplored. While NAFLD may contribute to baseline metabolic dysfunction and systemic inflammation [8,9], NAC can result in coagulopathy, hypoalbuminemia, and end-organ complications that increase surgical vulnerability [10-12].

Although several studies have examined perioperative outcomes in cirrhosis broadly [6,13], few have stratified outcomes by underlying etiology or disease stage in orthopedic populations. Moreover, data directly comparing NAFLD to NAC within the setting of CTR are lacking. Given the shift toward outpatient management and local anesthesia in CTR [14,15], understanding subtle differences in postoperative risk is critical for perioperative planning and patient counseling.

This study aims to compare complication rates following CTR in patients with NAFLD versus NAC. We hypothesize that patients with NAC will exhibit higher rates of postoperative complications, particularly infectious and renal sequelae, within both 90-day and one-year surveillance windows. Additionally, the economic implications of liver disease in carpometacarpal arthroplasty are poorly understood, and this study further aimed to evaluate differences in 90-day reimbursement between cirrhotic and non-cirrhotic NAFLD patients.

## Materials and methods

Data source and study design

This retrospective cohort study utilized the PearlDiver Mariner database (2010-2021), a national insurance claims dataset comprising over 122 million patients across private, Medicare, and Medicaid payers in the United States. The database enables longitudinal tracking of patients through Current Procedural Terminology (CPT) and International Classification of Diseases (ICD)-9/10 codes and is frequently used in orthopedic outcomes research. Given the use of deidentified claims data, this study was exempt from institutional review board approval.

All patients who underwent CTR were identified using CPT code 29848. To ensure complete longitudinal follow-up, only first-time CTR cases were included, with continuous database activity for at least six months before the index procedure and a minimum of two years postoperatively. This yielded an eligible cohort of 248,836 patients from an initial pool of 334,595 CTR cases. From this sample, patients with a preoperative diagnosis of NAC were identified using ICD-9 codes 571.5 and 571.6, and ICD-10 codes K743, K744, K745, K7460, and K7469. Patients with NAFLD were identified using ICD-10 codes K760 and K7581. Diagnoses were required to occur before the index CTR procedure to ensure appropriate classification of exposure status. This process yielded 1,516 patients with NAC and 6,427 with NAFLD who met all inclusion criteria.

Cohort selection and matching

To reduce selection bias and account for potential confounders, a one-to-one propensity score matching protocol was performed using a nearest-neighbor algorithm without replacement. Matching variables included age, gender, Charlson Comorbidity Index (CCI), and the presence of diabetes, osteoarthritis, obesity, tobacco use, alcohol abuse, and coagulopathy within one year preceding the index surgery. This yielded a final matched cohort of 642 patients with NAC and 642 patients with NAFLD, for a total study population of 1,284 patients.

Postoperative complications were categorized by time frame and analyzed at two intervals: within 90 days and within one year of the index surgery. Complications of interest included surgical site infection (SSI), deep vein thrombosis (DVT), pulmonary embolism, acute kidney injury (AKI), wound disruption, pneumonia, hematoma, urinary tract infection (UTI), transfusion, nerve injury, and cardiac arrest. Readmissions and reoperations were also assessed at both intervals, and a composite binary outcome labeled “any complication” was generated to capture the occurrence of one or more events. Missing data were handled intrinsically by PearlDiver through exclusion of patients without the required variables for cohort construction.

Outcome measures, adverse events, and statistical analysis

Baseline characteristics were compared using chi-square tests for categorical variables and Student’s t-tests for continuous variables. Multivariable logistic regression models were constructed to evaluate the association between hepatic disease subtype and each complication, adjusting for age, gender, and CCI. Odds ratios (ORs) with corresponding 95% confidence intervals (CIs) were reported. Statistical significance was set at a two-sided p-value <0.05. All analyses were performed within the PearlDiver platform using its integrated R-based statistical modules.

Cost analyses were performed using both parametric (t-test) and non-parametric (Wilcoxon rank-sum) methods to compare 90-day reimbursement amounts between matched cohorts. Reimbursement data were extracted from insurance claims, and total costs were calculated from the date of the index procedure through 90 days postoperatively.

## Results

Following inclusion and matching criteria, 642 patients with NAC and 642 patients with NAFLD were identified, yielding a final matched cohort of 1,284 patients. The two groups were well balanced in terms of age, gender, and CCI, confirming successful matching.

Baseline characteristics and comorbidity assessment

Deficiency anemia was more frequently observed in patients with cirrhosis (22.7%) compared to those with NAFLD (17.5%), representing 146 versus 112 patients, respectively (p = 0.022). Obesity was significantly more common in the NAFLD group, affecting 37.2% (239 of 642) of patients, compared to 31.5% (202 of 642) in the cirrhosis group (p = 0.034). There were no significant differences in the prevalence of other comorbid conditions, including diabetes (46.9 vs. 46.3), tobacco use (33.8% vs. 30.7%), alcohol abuse (9.5% vs. 9.7%), osteoarthritis (40.2% vs. 36.8%), or coagulopathy (7.8% vs. 9.0%) (Table 1).

**Table 1:** Baseline matched cohort demographics and comorbidities between NAFLD and NAC patients undergoing CTR. Reported numbers are represented as counts. Counts for age strata do not sum to the total cohort size due to PearlDiver programming censoring subgroup counts of fewer than 11 patients to protect privacy. Bold = statistically significant. NAC: non-alcoholic cirrhosis; NAFLD: non-alcoholic fatty liver disease; CTR: carpal tunnel release; COPD: chronic obstructive pulmonary disease; CKD: chronic kidney disease; CHF: congestive heart failure; CAD: coronary artery disease; HTN: hypertension; IHD: ischemic heart disease; PHD: pulmonary heart disease.

Variable	NAFLD (N = 642)	NAC (N = 642)	P-value
Age <55 years	179	179	-
Age >55 years	442	442	-
Male	185	185	-
Female	457	457	-
Asthma	106	95	0.443
COPD	112	96	0.256
CKD	63	68	0.712
CHF	43	44	1
CAD	104	91	0.351
Diabetes mellitus	301	297	0.867
HTN	418	388	0.094
IHD	102	94	0.587
Obesity	239	202	0.034
Osteoarthritis	258	236	0.228
PHD	1	3	0.617
Rheumatoid arthritis	33	36	0.805
Tobacco use	217	197	0.257
Alcohol abuse	61	62	1
Liver disease	407	423	0.381
Cancer	114	116	0.942
Coagulopathy	50	58	0.482
Deficiency anemia	112	146	0.022

Postoperative 90-day outcomes

Short-term postoperative complications were infrequent and generally comparable between groups. The incidence of acute kidney injury (AKI) within 90 days was 1.1% in both the NAFLD cohort cirrhosis cohort (7 of 642). Pneumonia occurred in 1.7% of cirrhosis patients (11 of 642) versus 1.1% of NAFLD patients (7 of 642), while urinary tract infections were observed in 5.0% and 5.3% of cirrhosis and NAFLD patients, respectively (32 vs. 34 patients). The overall composite complication rate at 90 days was 9.7% in the NAFLD group (62 of 642) and 10.6% in the cirrhosis group (68 of 642), a non-significant difference (adjusted OR = 0.82, 95% CI = 0.50-1.31, p = 0.420). Readmission rates at 90 days were also similar between NAFLD and NAC cohorts (3.4% vs. 3.3%) (Table 2).

**Table 2:** Complications within 90 days following CTR adjusted for age, gender, and CCI. Reported numbers are represented as counts. NAC: non-alcoholic cirrhosis; NAFLD: non-alcoholic fatty liver disease; CTR: carpal tunnel release; SSI: surgical site infection; DVT: deep vein thrombosis; AKI: acute kidney injury; UTI: urinary tract infection; CCI: Charlson Comorbidity Index; OR: odds ratio; CI: confidence interval

Adverse events	NAFLD (N = 642)	NAC (N = 642)	P-value	OR (95% CI)	Adjusted p-value
SSI	3	2	1	0.58 (0.03–4.33)	0.635
DVT	2	3	1	1.27 (0.16–8.25)	0.796
Wound disruption	2	4	0.682	0.40 (0.02–2.80)	0.421
Hematoma	2	2	1	N/A	N/A
Cardiac arrhythmia	0	0	1	N/A	N/A
Pneumonia	7	11	0.476	0.37 (0.06–1.45)	0.207
Transfusion	1	1	1	N/A	N/A
AKI	7	7	1	1.41 (0.35–5.07)	0.605
UTI	34	32	0.899	0.95 (0.49–1.76)	0.868
Reoperation	0	0	1	N/A	N/A
Readmission	21	22	1	0.87 (0.39–1.85)	0.734
Any complication	62	68	0.644	0.82 (0.50–1.31)	0.420
Nerve injury	1	1	1	N/A	N/A

Postoperative one-year outcomes

At one year, a statistically significant difference emerged in the incidence of acute kidney injury. Patients with non-alcoholic cirrhosis had an AKI rate of 6.2% (40 of 642), compared to 5.0% (32 of 642) in the NAFLD group. Multivariable logistic regression confirmed a lower likelihood of AKI in the NAFLD cohort (adjusted OR = 0.40, 95% CI = 0.16-0.88, p = 0.033) compared to the cirrhosis cohort. No statistically significant differences were found in other one-year outcomes, including pneumonia (4.4% vs. 3.3%), UTI (13.7% vs. 13.8%), or readmission (3.4% vs. 3.3%). The overall rate of any complication at one year was 22.1% (142 of 642) in the NAFLD group and 24.5% (157 of 642) in the cirrhosis group, which did not reach statistical significance (adjusted OR = 0.77, 95% CI = 0.54-1.09, p = 0.134) (Table 3).

**Table 3:** Complications within one year following CTR adjusted for age, gender, and CCI. Reported numbers are represented as counts. Bold = statistically significant. NAC: non-alcoholic cirrhosis; NAFLD: non-alcoholic fatty liver disease; CTR: carpal tunnel release; SSI: surgical site infection; DVT: deep vein thrombosis; AKI: acute kidney injury; UTI: urinary tract infection; CCI: Charlson Comorbidity Index; OR: odds ratio; CI: confidence interval

Adverse events	NAFLD (N = 642)	NAC (N = 642)	P-value	OR (95% CI)	Adjusted p-value
SSI	6	7	1	0.65 (0.09–2.88)	0.601
DVT	7	7	1	1.01 (0.21–3.91)	0.989
Pulmonary embolism	1	0	1	N/A	N/A
AKI	32	40	0.396	0.40 (0.16–0.88)	0.033
Cardiac arrhythmia	1	1	1	N/A	N/A
Wound disruption	3	6	0.504	0.32 (0.02–1.95)	0.296
Hematoma	2	3	1	N/A	N/A
Pneumonia	21	28	0.382	0.72 (0.31–1.52)	0.407
Transfusion	8	8	1	0.25 (0.01–1.43)	0.201
UTI	89	88	1	0.93 (0.61–1.39)	0.724
Readmission	21	22	0.497	0.87 (0.39–1.85)	0.734
Any complication	142	157	0.355	0.77 (0.55–1.08)	0.134
Nerve injury	1	1	1	N/A	N/A

Two-year reoperations

No significant differences were observed in reoperation rates or subsequent interventions, including revision CTR or manipulation under anesthesia. Reoperation at two years was rare in both groups and not statistically different in adjusted models.

Cost outcomes

Reimbursement data were available for 639 patients in the cirrhosis group and 641 in the NAFLD group. The mean 90-day reimbursement was higher for patients with cirrhosis ($5,860.34 ± 9,792.72) compared to those with NAFLD ($5,488.57 ± 6,420.22), though this difference was not statistically significant on two-sample t-testing (p = 0.564). The median reimbursement was $3,610.00 in the cirrhosis group and $3,820.00 in the NAFLD group (p = 0.178, Wilcoxon rank-sum). Total reimbursement across the groups was $3,744,759.00 and $3,518,175.00, respectively (p = 0.615) (Table 4).

**Table 4:** 90-day reimbursement outcomes following CTR in patients with cirrhosis versus NAFLD. P-values were calculated using a two-sample t-test for mean, the Wilcoxon rank-sum test for median, and total reimbursement. NAC: non-alcoholic cirrhosis; NAFLD: non-alcoholic fatty liver disease; CTR: carpal tunnel release

Outcome	Cirrhosis (n = 639)	NAFLD (n = 641)	P-value
Mean cost, USD (SD)	$5,860.34 (±9,792.72)	$5,488.57 (±6,420.22)	0.564 (t-test)
Median cost, USD	$3,610.00	$3,820.00	0.178 (Wilcoxon)
Total reimbursement, USD	$3,744,759.00	$3,518,175.00	0.615 (Wilcoxon)

## Discussion

This matched cohort study demonstrates that while 90-day outcomes following CTR are comparable between patients with NAFLD and NAC, the risk of AKI at one year is significantly elevated in the NAC cohort. Our analysis also revealed that patients with NAC incurred higher total 90-day reimbursements compared to patients with NAFLD/NASH, though this difference did not reach statistical significance. This trend may reflect increased resource utilization in patients with underlying hepatic dysfunction, warranting further investigation in larger cohorts.

Although absolute rates were low, there was a 24% relative increase in AKI among cirrhotic patients at one year compared to NAFLD patients. However, these findings may be due to several mechanisms underlying higher AKI risk in cirrhosis, including systemic vasodilation, impaired renal autoregulation, and the hepatorenal syndrome, rather than postoperative complications [11,16,17]. Additionally, the use of ICD codes for identifying complications such as AKI may result in detection bias in cirrhotics, as these patients undergo more intensive laboratory surveillance.

Our findings also underscore the distinction between NAFLD and NAC in orthopedic risk stratification. While NAFLD is associated with systemic inflammation and insulin resistance [8,9], its impact on CTR outcomes appears minimal, consistent with prior orthopedic literature discussing no significant difference in patient-reported outcomes (PROs) from insulin resistance/diabetes mellitus [18].

Importantly, no increased risk was observed for common short-term complications, such as wound infection, DVT, or reoperation, in either group. These results support the general safety of CTR in both hepatic disease states when appropriate preoperative screening and risk modification are performed [19,20].

Our study contributes to a growing body of literature advocating for procedure-specific risk assessment rather than blanket risk assumptions based on chronic disease [21]. The nuanced difference in AKI risk suggests that surveillance and fluid management strategies may warrant closer attention in patients with NAC, even in minor surgical settings [16,22,23].

Limitations

This study is subject to several limitations inherent to retrospective database analyses. First, the PearlDiver platform relies on administrative claims data, which may be vulnerable to miscoding, underreporting, or misclassification of diagnoses and outcomes. Second, granular clinical variables such as liver function tests, albumin levels, Model for End-Stage Liver Disease scores, or Child-Pugh class were not available, limiting our ability to stratify cirrhosis severity or adjust for laboratory-confirmed liver dysfunction. Third, we were unable to assess functional outcomes such as symptom relief, return to work, or hand strength, which may be more relevant to CTR-specific recovery. Fourth, residual confounding may persist despite robust one-to-one matching and multivariable adjustment. Fifth, although the analytic approach is reproducible within PearlDiver, the use of platform-based query tools limits the direct transferability of individual code lists to alternative external datasets. Finally, findings from this insured U.S. population may not generalize to underinsured or global populations, and the rarity of complications such as reoperation limits the power to detect small but clinically important differences.

## Conclusions

Patients with NAC undergoing CTR do not appear to experience higher short-term complication rates compared to those with NAFLD. Our results did show that cirrhotic patients may be at increased risk of delayed renal complications, such as AKI, at one year, but this may be attributed to the hepatorenal syndrome they experience as a result of their liver pathology. These findings suggest that hepatic disease severity may not need to be considered in risk assessment when comparing NAFLD and cirrhosis, supporting similar postoperative monitoring following CTR.
